# Autoimmune Encephalitis in Long-Standing Schizophrenia: A Case Report

**DOI:** 10.3389/fneur.2021.810926

**Published:** 2022-02-11

**Authors:** Amy Vaux, Karen Robinson, Burcu Saglam, Nathan Cheuk, Trevor Kilpatrick, Andrew Evans, Mastura Monif

**Affiliations:** ^1^Department of Neurology, Royal Melbourne Hospital, Melbourne, VIC, Australia; ^2^Department of Psychiatry, Royal Melbourne Hospital, Melbourne, VIC, Australia; ^3^Florey Institute of Neuroscience and Mental Health, The University of Melbourne, Melbourne, VIC, Australia; ^4^Department of Neuroscience, Monash University, Melbourne, VIC, Australia

**Keywords:** autoimmune encephalitis, NMDA encephalitis, chronic schizophrenia, diagnostic dilemma, neuropsychiatric disorders, case report, NMDA antibody

## Abstract

Anti-N-methyl-D-aspartate (NMDA) receptor antibody (anti-NMDAR Ab)-mediated encephalitis is an autoimmune disorder involving the production of antibodies against NMDARs in the central nervous system that leads to neurological or psychiatric dysfunction. Initially described as a paraneoplastic syndrome in young women with teratomas, increased testing has found it to be a heterogeneous condition that affects both the sexes with varying clinical manifestations, severity, and aetiology. This case report describes a 67-year-old man with a 40-year history of relapsing, severe, treatment-refractory schizophrenia. Due to the worsening of his condition during a prolonged inpatient admission for presumed relapse of psychosis, a revisit of the original diagnosis was considered with extensive investigations performed including an autoimmune panel. This revealed anti-NMDAR Abs in both the serum and cerebrospinal fluid on two occasions. Following treatment with intravenous immunoglobulin and methylprednisolone, he demonstrated rapid symptom improvement. This is a rare case of a long-standing psychiatric presentation with a preexisting diagnosis of schizophrenia subsequently found to have anti-NMDAR Ab-mediated encephalitis. Whether the case is one of initial NMDAR encephalitis vs. overlap syndrome is unknown. Most importantly, this case highlights the need for vigilance and balanced consideration for treatment in cases of long-standing psychiatric presentation where the case remains treatment refractory to antipsychotics or when atypical features including seizures and autonomic dysfunction or focal neurology are observed.

## Introduction

Anti-N-methyl-D-aspartate (NMDA) receptor (NMDAR) antibody (Ab)-mediated encephalitis was first described in 2005 as a neurological paraneoplastic syndrome occurring in women presenting with psychiatric disturbance and teratomas (1). This led to the identification of a range of anti-NMDAR Abs with varying immunoglobulin (Ig) subclasses against NR1 and NR2 subunits of the NMDAR, in patients with numerous clinical syndromes as well as in asymptomatic healthy controls (2–5). However, it is the immunoglobulin G (IgG) anti-NR1 antibodies in the cerebrospinal fluid (CSF), with a predefined set of symptoms that are diagnostic for the syndrome (6). In the CNS, IgG NR1 antigen-antibody binding results in reversible titratable internalisation of the NMDAR that reduces NMDA signalling and results in the symptom development. Antibody presence in the serum alone is not sufficient to cause symptoms (3, 4, 7). However, processes disrupting the blood–brain barrier can allow antibody circulation within the CSF, leading to antigen–antibody binding (4).

The aetiology is attributed to tumours in 38% of cases of which teratoma is the most common type (8). Anti-NMDAR Ab-mediated encephalitis has also been associated with infective encephalitides, including herpes simplex virus (HSV) encephalitis and cryptococcal meningitis, with neuroinflammation possibly acting as a mechanism triggering anti-NMDAR Ab production (9–15).

Anti-NMDAR Ab-mediated encephalitis typically follows a multiphasic course presenting with a flu-like prodrome prodromal illness followed by psychiatric symptoms of delusions, hallucinations, agitation, thought disorder, catatonia and sometimes even coma (16, 17). Later, neurological symptoms such as seizures (70% of cases) or autonomic dysfunction (hyperthermia, hypertension, and hypersalivation) may also occur (16–21). Common investigations in addition to antibody testing include electroencephalograms (EEGs) with abnormalities in 80% of patients and the pathognomonic “extreme delta brush” pattern in 30–58% of cases, MRI that is transiently abnormal in 33–50% of patients, and ^18^F-Fluorodeoxyglucose-PET (FDG-PET), which show focal or multifocal abnormalities in ~70% of cases (8, 16, 18, 20, 22–28).

Relapse has been shown to occur in 12–24% of patients with anti-NMDAR Ab-mediated encephalitis, with a relapse up to 13 years postfirst episode of encephalitis reported (8, 29, 30). Relapse risk is increased in patients with non-tumor and those who do not receive immunotherapy (8, 29, 30). The majority of literature discussing cases of anti-NMDAR Ab positivity has focused on either acute encephalitis or primary psychiatric presentations (8, 31). There are limited examples of cases reported of chronic presentations or those with prolonged latency between symptom onset to diagnosis, though anecdotally we are aware of cases with intervals of up to 10 years. Those with chronic psychiatric diagnoses form a separate category where it can be difficult to ascertain if the psychiatric diagnosis still stands or if the underlying aetiology is that of an autoimmune condition.

We present a case of a man with a long-standing psychiatric diagnosis where anti-NMDAR Abs were subsequently found in the CSF and serum. This case highlights the need for clinical vigilance in not only first-presentation psychosis, but also in those with a long-standing history of psychiatric issues where an autoimmune entity could be at play.

## Case Description

A 67-year-old man with a 40-year history of schizophrenia was admitted to an inpatient psychiatric unit in January 2021 with a relapse of psychosis. His mental state deterioration followed the recent death of his mother and demonstrated increasing paranoia and thought disorder resulting in social withdrawal.

He was first diagnosed with schizophrenia at the age of 25 years following the development of acute psychosis characterised by paranoid delusions, thought disorder, and auditory hallucinations. Though this was a *de novo* illness, it had been preceded by premorbid loner behaviours and potential schizoid personality traits. Over the next 20 years, he relapsed every 3–4 years with episodes characterised by prolonged, treatment-refractory events of highly disorganised and fluctuating psychosis. In several of these relapses, he required electroconvulsive therapy (ECT), which he responded well to.

After a 10-year period of being lost to follow-up between 1999 and 2010, he presented to the emergency department in early 2011 with a psychotic relapse and a first reported generalised seizure. A brain CT scan showed a small old left caudate infarct and an EEG performed was normal. A similar episode occurred in 2015 where a second tonic-clonic seizure occurred, and he again was diagnosed as having had a psychiatric relapse and subsequently had a prolonged inpatient psychiatric admission. Following a protracted psychiatric episode in 2018 that failed to respond to the psychotropic treatment, he underwent 9 sessions of ECT that appeared to reduce some of his psychiatric symptoms. During these relapses, he demonstrated substantial cognitive decline. In between episodes, he maintained a relatively high functioning baseline (see Appendix 1) whilst on antipsychotics although he was unable to participate in paid employment. Prior to the 2021 relapse, he was living independently in the community and in previous periods had been a carer for his late mother.

On admission to the psychiatric unit in January of 2021, his symptoms were characterised by thought disorder and hypomania. His affect and behaviour were highly labile demonstrating perceptual disturbances (auditory and visual) and psychomotor agitation, occasionally physical aggression towards staff and later appearing lethargic, dazed, and withdrawn. Moreover, he had multiple low-grade fevers and developed several autonomic and dyskinetic symptoms including hypersalivation, speech disturbance, and unsteady, widened shuffling gait. These symptoms were not correlated with medication changes, but appeared to spontaneously fluctuate (Table 1).

**Table 1:** Inpatient unit (IPU) admission summary.

**Timeline**	**Event/Assessment and medication changes**
D1 Admission	Mental State Examination (MSE)^a^: psychotic with prominent thought disorder and hypomanic symptoms. Noted unusual wide based quick stepping gait. Admission medications: Olanzapine 10 mg three times a day, Atorvastatin 40 mg daily, Thiamine 100 mg daily Aspirin 100 mg daily, Perindopril 8 mg daily, Amlodipine 10 mg daily, Monoxidine 400 mg morning, and 200 mcg at night.
D10	Appearing improving affect, denying perceptual disturbance, stabilising without change to psychotropic regimen
D11	Cognitive assessments completed: MoCA^b^ 23/30
D17	Requiring excessive reassurance from staff, thought disordered but no psychomotor agitation or perceptual disturbance. Alteration to olanzapine from 10 mg three times a day to 10 mg in the morning, 25 mg at night.
D18	Start of slurred speech development, significantly labile affect
D23	Low-grade fever, 37.7, No infective symptoms
D26	Mild sore throat. Isolated and COVID swab sent, returning negative.
D27	Commenced new Lorazepam 1 mg twice daily to assist with agitation
D29	2nd low-grade fever 37.7. No infective symptoms. Creatine Kinase (CK) tested returning 65 u/L (reference range 40–200)
D31	Lithium dose increased to 250 mg in the morning/375 mg at night. Sodium valproate 400 mg at night commenced.
D36	Sodium valproate dose increased from 400 mg at night to 200 and 400 mg at night
D38	MSE AM: sedated, slurred speech, hypersalivating, unsteady slightly wide based gait MSE PM: highly driven with significant psychomotor agitation, limited sleep
D41	Repeat MOCA 16/30
D42	Morning lithium dose further reduced to 125 mg
D45	MSE: ongoing prominent thought disorder and slurred speech but improving agitation
D46	3rd low grade fever to 37.5, however MSE improving, more settled, less irritable.
D52	MSE deterioration: increased agitation, irritability, and thought disorder. Recurrence of auditory perceptual disturbance and cognitive deterioration.
D55	Acute worsening of agitation with physical aggression and sexual inhibition. Commenced cross titration of olanzapine to Paliperidone.
D59	4th low-grade temperature to 37.8 degrees. No infective symptoms
D66	Ongoing highly fluctuating symptoms. Paliperidone increased to 6 mg, olanzapine weaning.
D72	Repeat MOCA 20/30
D82	Repeat MOCA 15/30
D90	Affect improving but fluctuations ongoing in cognition. Sodium valproate dose weaning commenced Paliperidone increased to 9 mg.
D101	Behaviours worsening with aggression, impulsivity. Noted auditory and visual perceptual disturbances and prominent thought disorder. Slurred speech again noted. Paliperidone dose reduced. Delirium screen including CT brain completed with no new findings.
D123	Transferred to acute medical hospital. MSE on arrival: flattened affect with slurred mumbling speech. Labile behaviours from withdrawn, sedated, quiet, to later significant psychomotor agitation, aggression towards staff. Noted hypersalivation, shuffling wide-based gait. Psychotropic medications on admission to Acute Medical Hospital: Paliperidone 6 mg at night, Lithium 375 mg at night, Lorazepam 1 mg at night and 1 mg in the morning as needed, Zopiclone 7.5 mg at night. This medication regimen had been stable for several weeks and did not change during his Tertiary Medical Hospital Admission.

a *Mental State Examination (MSE): A structured examination approach that assesses patients' appearance, behaviour, speech, mood and cognition (32)*.

b *Montreal Cognitive Assessment (MoCA): a clinician administered 30 questions designed to detect and monitor cognitive impairment (33)*.

Due to ongoing fluctuations in his psychiatric and behavioural symptoms with no improvement in trend, the decision was made for referral to the tertiary medical hospital site to undergo more extensive investigation for reconsideration of diagnosis.

Haematological, biochemical, and septic screens were performed and yielded unremarkable results (Table 2). An EEG was normal and a CT brain demonstrated no acute findings, noting only the known small old left caudate infarct. Microbiological and biochemical analysis of a CSF sample returned results within normal limits. However, his serum and CSF returned positive tests for anti-NMDAR Abs.

**Table 2:** Results table.

**Test**	**Result**	**Test**	**Result**
Anti-NMDAR Antibodies^*^	CSF 09/06/21 detected CSF 12/7/21 Detected (no discernible difference in staining intensity) Serum 24/6/21 detected	CSF 12/7/21	Protein 0.39 (ref <0.45 g/L), glucose 3.6 Erythrocytes 74, polymorphs 0, lymphocytes 2 No oligoclonal IgG bands Culture negative Cryptococcal antigen negative
Voltage Gated Potassium Channel Antibodies	Not detected	CSF 9/6/21	Protein 0.31 (<0.45g/L) glucose 3.7 Erythrocytes 0, Polymorphs 0, lymphocytes 0
AMPA receptor antibodies	Not detected	Viral CSF PCR	Serum HSV1 IgG positive, IgM negative CSF HSV 1/2, VZV, CMV, Adenovirus no detected
Neuronal antibodies	Negative	Alpha-feto protein	Not elevated
Mitochondrial antibodies	Not detected	Syphilis serology	Negative
HIV serology	Negative	Hepatitis B + C serology	Negative
CASPR2 antibodies	Not detected	CMV serology	IgG positive, IgM negative
Non-contrast MRI brain 19/07/21	No acute intracranial pathology.• In particular no imaging features of autoimmune encephalitis.	CT chest, abdomen, pelvis 14/07/21	No evidence of solid malignancy. Long-standing occlusion of infrarenal abdominal aorta.
Non-contrast MRI brain 19/07/21	No acute intracranial pathology.• In particular no imaging features of autoimmune encephalitis.	PET 22/07/21	No FDG evidence of malignancy. Increased metabolism of the parietal, temporal and to lesser frontal cortices. Although non-specific, this is reported as a finding seen in anti-NMDA-receptor encephalitis.

*CSF, cerebrospinal fluid; HSV, herpes simplex virus; VZV, varicella zoster virus; CMV, cytomegalovirus*.

* *Antibody testing was carried out by Pathology Queensland (NATA/RCPA Corporate Accreditation Number 2639) with Health Support Queensland. Anti-NMDA-receptor IgG antibodies were detected in serum and cerebrospinal fluid (CSF) by indirect immunofluorescence using a commercial assay containing four biochips of primate hippocampus, primate cerebellum, fixed NR1-transfected human embryonic kidney 293 (HEK293) cells, and fixed non-transfected control HEK293 cells (IIFT: Glutamate Receptor Mosaic 3, Euroimmun, Lübeck, Germany, UK) (34, 35). When the second sample was received the CSF was tested in parallel with the earlier CSF specimen with no discernible difference in intensity of the staining identified. Dilution base or titre testing is not available in Australian laboratories*.

Due to concerns of acute behaviour exacerbation, particularly with uncertainties regarding the clinical significance of the positive antibodies, it was felt that methylprednisolone pulsing would be inappropriate. In preference, he completed a 5-day course of 0.4 mg/kg/day of intravenous Ig (IVIg) (days 145–150 of his combined psychiatric and medical inpatient admissions).

For the following 7 days, his mental state remained persistently labile with ongoing thought disorder and agitation. However, on days 9–12 post-IVIg, there was marked, sustained improvement in his mental state. On review day 14 post-IVIg, he was observed to be lucid with a reactive affect, speaking and behaving politely, and demonstrating both capacities to understand medical discussions and recall conversations.

Computed tomography chest, abdomen, and pelvis and PET scan, MRI brain, and second lumbar puncture day 12 post-IVIg failed to identify a paraneoplastic source of NMDAR antigens such as a teratoma or any neurodegenerative disorders. His second CSF sampling day 12 post-IVIG again returned positive for NMDAR Abs.

He later underwent a 5-day course of 500 mg daily IV methylprednisolone as it was felt his mental state had improved enough to be safe for a trial of steroid therapy and that this would align with the more aggressive treatment approach increasingly recommended in the literature (26, 36). This was tolerated well and he continued small progressive daily improvements in his mental state and cognition.

He was then discharged home postcommencement of rituximab, with the plan to continue rituximab on a 6-monthly basis. At outpatient review 6 months posttreatment, he maintained his improved psychiatric state and is living independently in the community. He reported no hallucinations or delusions and denied any new neurological symptoms. He reported improvements in his memory. He was continued on the same psychiatric drug regimen. However, his stability has meant the psychiatric team has started weaning his lorazepam and assuming ongoing stability with treatment on rituximab, there may be scope to further reduce his antipsychotics. As it is impossible to determine when he developed the anti-NMDAR Ab, ongoing management must be considered for the likelihood of an overlap syndrome of a background schizophrenia with superimposed anti-NMDAR Ab-mediated encephalitis, both now well-controlled with antipsychotic therapy and rituximab.

## Discussion

This is an unusual case of anti-NMDAR Ab-mediated encephalitis in a patient with a preexisting diagnosis of chronic atypical treatment-refractory schizophrenia. This case fulfils all the diagnostic criteria for anti-NMDAR Ab-mediated encephalitis:

1. Presence of NMDAR antibodies in CSF: *This case demonstrated positive NMDAR Abs in CSF sampling on two separate occasions*.
2. Consistent clinical symptoms: *This case demonstrated disorganised psychiatric and behavioural disturbance, speech disturbance (mumbling, non-sensical), hypersalivation, and intermittent fevers. These were not correlated with medication changes*.
3. Treatment response: *This case demonstrated acute improvement in behaviour, orientation, resolution of autonomic dysfunction, and hypersalivation in appropriate timeframe post-IVIg. This acute improvement was out of keeping with his pattern of symptom recovery in previous psychiatric relapse* (6).

What remains ambiguous is whether his current and past psychiatric disturbance are explainable by the anti-NMDAR Ab-mediated encephalitis alone or whether it reflects an anti-NMDAR Ab encephalitis superimposed on a prior schizophrenia. It is impossible to retrospectively determine when he developed the anti-NMDAR Abs and there is always the possibility of diagnostic assay error. However, the reproducibility of the anti-NMDAR Ab in his CSF on two occasions adds validity to the diagnosis, as studies demonstrating the specificity of CSF antibodies when applied to general and psychiatric patient groups, unlike serum testing (3, 37, 38).

This case may represent sporadic anti-NMDAR Ab-mediated encephalitis developing in 2021 mimicking his typical psychiatric episodes. Alternatively, this may represent a long-term relapsing-remitting anti-NMDAR Ab-mediated encephalitis, where the lack of appropriate treatment has led to prolonged medication-resistant episodes. This would be supported by his previous responsiveness to ECT, which is known to be efficacious in anti-NMDAR Ab-mediated encephalitis in addition to treatment-refractory psychotic illnesses (39–41). Moreover, this would explain as to his fluctuating disease course as well as the two previous episodes of seizures in 2011 and 2015.

Anti-NMDAR Ab-mediated encephalitis was only identified 15 years ago and most cases involve short periods (weeks to months) between symptom onset and diagnosis. Examples of prolonged symptom onset to diagnosis periods are rare and case descriptions are limited. This case raises the possibility of a group of patients with long-standing relapsing-remitting anti-NMDAR Ab-mediated encephalitis who remain undiagnosed as their symptoms developed prior to when the condition was first identified. Additionally, the difficulty in distinguishing anti-NMDAR encephalitis symptoms from psychiatric psychosis supports the hypothesis of NMDA deregulation as a contributing mechanism in the development of schizophrenia.

In cases involving long-standing diagnoses, clinicians are prone to diagnostic error from premature closure bias and subsequently exclude explanations that do not align with their current beliefs. This case highlights the importance of recognising red flags such as neurological symptoms, autonomic disturbances or failure to respond to appropriate antipsychotic treatment in the treatment of patients with psychiatry. The decision for invasive investigations requires a high index of clinical suspicion to justify. However, by nature of their symptoms, this patient group may be unreliable historians, and as such rigorous history can be limited unless substantive collateral history is available. Moreover, with prolonged hospitalisation superimposed disorders such as delirium, or complications such as neuroleptic malignant syndrome, can act as confounders in clinical assessment (42). However, conclusive diagnosis remains challenging due to the pervasive risk of false positives and negatives, even with the gold standard investigation with CSF testing.

Seeking medical consultation for reconsideration of diagnosis and completion of the extensive investigation were ultimately judicious decisions by the psychiatric and neurology teams that proved critical for his treatment and ongoing care.

## Conclusion

Further research is needed to guide if screening for anti-NMDAR Ab-mediated encephalitis should be considered for patients with long-standing treatment-refractory schizophrenia with atypical symptoms or syndromes. Clinicians must balance the risks of invasive investigation and acute- and long-term side effects of treatment, against the potential of an alternate diagnosis and effective treatments. This case highlights the importance of maintaining clinical vigilance in patients who fail to respond to treatment and the pervasive risk of diagnostic error through premature closure bias.

## Data Availability Statement

The original contributions presented in the study are included in the article/Supplementary Material, further inquiries can be directed to the corresponding author/s.

## Ethics Statement

Ethical review and approval was not required for the study on human participants in accordance with the local legislation and institutional requirements. The patients/participants provided their written informed consent to participate in this study. Written informed consent was obtained from the individual(s) for the publication of any potentially identifiable images or data included in this article.

## Author Contributions

AV prepared the first draft of the manuscript with a contribution from BS who provided a summary of the most recent psychiatric inpatient stay of the individual. AV, KR, BS, NC, TK, AE, and MM were all involved in the clinical care of the patient and critically reviewed the manuscript drafting for its factual accuracy, assessments, and conclusions. All authors provided approval for the final manuscript.

## Funding

Funds to assist in the publication of this case report have been taken from a National Health and Medical Research Grant MRF1201062 provided to MM.

## Conflict of Interest

The authors declare that the research was conducted in the absence of any commercial or financial relationships that could be construed as a potential conflict of interest.

## Publisher's Note

All claims expressed in this article are solely those of the authors and do not necessarily represent those of their affiliated organizations, or those of the publisher, the editors and the reviewers. Any product that may be evaluated in this article, or claim that may be made by its manufacturer, is not guaranteed or endorsed by the publisher.
