# Supplementary material for: Autoimmune Encephalitis in Long-Standing Schizophrenia: A Case Report
Source: Front Neurol. 2022 Feb 11;12:810926. doi: 10.3389/fneur.2021.810926 (PMC8873086; doi:10.3389/fneur.2021.810926)
Supplement: Supplementary file 1 [file Data_Sheet_1.pdf]

Appendix

Appendix 1 Past admission summary

Note: due to presentations to alternative health services, only limited records between 1980-1997 could be obtained.

**Appendix 1: Past Psychiatric Admission Summary**	
Year	Events
Jan - March 1978	First episode of psychosis: admitted to inpatient psychiatric unit 30/1/78 - 6/2/78 (discharged against medical advice at father's request), re-admitted 10/2/78 - 30/3/78.<br><br>History of presenting complaint: returned from work one evening with delusions of grandiosity and paranoia. Thought disorder with loosening of associations, thought blocking, ideas of reference. Auditory hallucinations.<br><br>Diagnosed with acute schizophrenia.<br><br>Failure to respond to medication management, given 7 x sessions of electroconvulsive therapy (ECT).<br><br>Medications on discharge: Thioridazine 200mg twice daily, amitriptyline 75mg twice daily.
April 1978	Outpatient review noted mental state was stable, had self-ceased medication following discharge however had since developed tremors, stiffness and gait disturbance. Trial of Benztropine provided. Progressive improvement in symptoms, Thioridazine re-started later.
January 1995	Inpatient Unit admission for psychosis, noted odd posturing and mannerisms. Commenced on haloperidol and Benztropine
December 1997	Inpatient unit admission for psychosis following non-adherence to medications for 5 months
April 2011	Increased alcohol consumption Jan 2011 aligned with deterioration in mental state: increasing symptoms of hypomania then progressive psychosis.<br><br>Admitted to hospital when witnessed tonic clonic seizure with urinary incontinence, and Todd's paresis following. CT-brain showing old small L) caudate infarct and infective screens though hypertensive. Found to be floridly psychotic when conscious level improved.<br><br>Inpatient Unit Admission with severe psychosis with poor medication response. Underwent course of ECT. Discharged with 15mg olanzapine twice daily, Sodium Valproate 1500mg at night, and Quetiapine 50mg twice daily.

October 2015	Found on footpath with blood from head and noted urinary incontinence. Then witnessed tonic clonic seizure when ambulance arrived. Hypertensive and blood sugar 6.8. Poor recall of events. CT-brain and serum screens returning unremarkable results. Discharged after brief medical admission.
November 2015	Inpatient Unit admission for psychotic relapse of chronic schizophrenia due to medication non-adherence. On admission demonstrating blunted affect, tangentiality, poor self-care, lip-smacking and masticatory movements. Medication regimen including olanzapine 10mg three times a day, Quetiapine 100mg at night, Sodium valproate 700mg twice daily. Hypertensive throughout admission. NuCog score by time of discharge 95.5.
July 2018	Inpatient Unit admission for acute psychosis with paranoia, aggression, thought disorder. Clinician note indicating that his prolonged psychosis admissions often raised concerns for underlying delirium but is consistent with his usual pattern that takes months to resolve. No significant medication changes attempted, given 9 sessions of ECT. NuCOG[2] when psychotic 60.5. Frequent issues with incontinence associated with fluctuations of symptoms.<br><br>Discharge medications: olanzapine 10mg morning 20mg at night, Quetiapine 100mg at night, Lithium 250mg twice daily. Persistent hypertension during admission.
	1. Acronyms: Electroconvulsive therapy (ECT)<br>2. NuCOG Score: the Neuropsychiatry Unit Cognitive Assessment Tool (NUCOG) is a validated cognitive screening tool used in psychiatric, neurological, and neurodegenerative disorders. (1)

Note on Hypertension:

Hypertension was noted to be a long-standing issue throughout his admissions between 2011 and 2021, requiring multiple anti-hypertensives and still limited control achieved.

Changes in his blood pressure not associated with symptom fluctuation. CT Chest/ abdomen/ pelvis completed in July 2021 found long-standing occlusion of the infrarenal abdominal aorta to the proximal common iliac arteries. Due to this he had poor flow within his left renal artery (distal to occlusion) and atrophy of the left kidney. Significant vascular disease with renal artery involvement is likely the cause of his treatment refractory hypertension.

Additional Psychiatric History:

Childhood and development: normal pregnancy and developmental milestones, middle of 3 boys with supportive upbringing.

Good performance in primary school, active in sport. He became more independent and isolated in high school, occasionally demonstrating ‘loner’ behaviours, but he maintained good school performance. Commenced tertiary degree but dropped out after a few years in favour of full time work.

Drugs and alcohol: occasional marijuana in early 20s, nil other drug use. A single period of sustained heavy alcohol use occurred 2011. There was no confirmed heavy alcohol consumption thereafter. However, the family did raise concerns that concealed alcohol consumption could be possible.

Social situation prior to admission: living in a privately owned unit. He was active in visiting his mother in her nursing home daily. Financially supported by disability pension.

References

1. Walterfang M, Siu R, Velakoulis D. The NUCOG: validity and reliability of a brief cognitive screening tool in neuropsychiatric patients. Aust N Z J Psychi
